# Long Term Results of Anterior Corpectomy and Fusion for Cervical Spondylotic Myelopathy

**DOI:** 10.1371/journal.pone.0034811

**Published:** 2012-04-13

**Authors:** Rui Gao, Lili Yang, Huajiang Chen, Yang Liu, Lei Liang, Wen Yuan

**Affiliations:** Department of Spine Surgery, Changzheng Orthopedic Hospital, Second Military Medical University, Shanghai, China; University of Louisville, United States of America

## Abstract

**Background:**

Results showed good clinical outcomes of anterior corpectomy and fusion (ACCF) for patients with cervical spondylotic myelopathy (CSM) during a short term follow-up; however, studies assessing long term results are relatively scarce. In this study we intended to assess the long term clinical and radiographic outcomes, find out the factors that may affect the long term clinical outcome and evaluate the incidence of adjacent segment disease (ASD).

**Methods:**

This is a retrospective study of 145 consecutive CSM patients on ACCF treatment with a minimum follow-up of 5 years. Clinical data were collected from medical and operative records. Patients were evaluated by using the Japanese Orthopedic Association (JOA) scoring system preoperatively and during the follow-up. X-rays results of cervical spine were obtained from all patients. Correlations between the long term clinical outcome and various factors were also analyzed.

**Findings:**

Ninety-three males and fifty-two females completed the follow-up. The mean age at operation was 51.0 years, and the mean follow-up period was 102.1 months. Both postoperative sagittal segmental alignment (SSA) and the sagittal alignment of the whole cervical spine (SACS) increased significantly in terms of cervical lordosis. The mean increase of JOA was 3.8±1.3 postoperatively, and the overall recovery rate was 62.5%. Logistic regression analysis showed that preoperative duration of symptoms >12 months, high-intensity signal in spinal cord and preoperative JOA score ≤9 were important predictors of the fair recovery rate (≤50%). Repeated surgery due to ASD was performed in 7 (4.8%) cases.

**Conclusions:**

ACCF with anterior plate fixation is a reliable and effective method for treating CSM in terms of JOA score and the recovery rate. The correction of cervical alignment and the repeated surgery rate for ASD are also considered to be satisfactory.

## Introduction

Cervical spondylotic myelopathy (CSM) is the most common cause of spinal cord dysfunction in persons more than 55 years of age [1]. With aging, the degenerative changes in the cervical spine, including degeneration of the intervertebral discs, ligaments and connective tissue of the cervical vertebrae, can cause compression of the spinal cord, which results in myelopathy. Patients with CSM may have various symptoms including sensory and motor disturbances, but the onset of myelopathy is usually insidious [2]. Treatment of patients with CSM depends on the type and severity of neurologic symptoms, as well as signs and progression of the disease. Conservative treatment is recommended for patients without the presence of neurologic findings, while surgical decompression is recommended for patients with progressive disease, severe symptoms, or symptoms severely affecting daily activities [3].

Anterior approaches for the surgical treatment of CSM, including anterior cervical discetomy and fusion (ACDF) and anterior cervical corpectomy and fusion (ACCF), have been widely used in the past 50 years [4], [5]. ACCF provides improved visualization over ACDF in the removal of the osteophytes and ossified posterior longitudinal ligament (OPLL) [6]. Previous results showed good clinical outcomes of ACCF for CSM in a short term follow-up [7], [8]. Although the long term results were also satisfactory in some reports, studies assessing long term results of ACCF for CSM are relatively scarce [9], [10].

We conducted a retrospective study of a series of consecutive patients who underwent ACCF for the treatment of spondylotic myelopathy with a minimum 5-year follow-up. The goal of this study was to assess the long term clinical and radiographic outcomes, find the factors that may affect the long term clinical outcome and evaluate the incidence of adjacent segment disease (ASD).

## Materials and Methods

### Patient Selection and Surgical Technique

This study was approved by the Institutional Review Broad of Second Military Medical University, Shanghai, China. Between January 1, 1996 and June 30, 2006, 303 patients underwent anterior decompression and fusion for cervical spondylotic myelopathy by the senior surgeon (W.Y.). Patients with cervical spine trauma, neoplasm, infection, congenital deformations, previous cervical spine surgery and chronic systemic illnesses such as rheumatoid arthritis and neurodegenerative diseases were excluded in this study.

All patients were clinically and radiographically evaluated before surgery. Clinical evaluation consisted of medical history and physical examination (neurologic examination included). The clinical results were assessed with the Japanese Orthopedic Association (JOA) scoring system for cervical myelopathy [11]. Standard anterior–posterior, lateral and flexion-extension X-rays, magnetic resonance imaging (MRI) and computed tomography (CT) of the cervical spine were conducted as radiological evaluation. ACCF was performed using a standard channel technique as previously described, iliac-crest bone autograft or titanium mesh cage (TMC) (Sofamor Danek; Depuy) filled with local autograft bone from the vertebral body was implanted after corpectomy. Moreover, segmental fixation was performed with anterior cervical plates (Zephir and Orion, Sofamor Danek; Coddman and Slimlock, Depuy) after decompression and fusion [12].

### Postoperative Follow-Up

All patients were requested to come back for a routinely examination at 3 months, 6 months and 12 months after the operation, then at irregular intervals depending on their clinical status and suggestions of the senior surgeon. These patients received invitations for clinical and radiologic examinations between June and November 2011. The examinations were performed by investigators who had no therapeutic relationship to the individual patient. A total of 127 patients were lost to follow-up due to change of address or phone number, 23 patients refused to return because of the long distance and old age, or the feeling that they did not have any significant problems that required a visit, 7 patients died of unrelated causes, 1 patient unfortunately had stomach cancer and was too ill to return. In the end, a total of 145 patients responded to the invitation and made return visits. The informed consents were obtained from every participant. The consents were in written form and the consent procedure was approved by ethics committees.

During the follow-up, clinical examination was assessed by using the JOA scoring system [11]. The JOA recovery rate proposed by Hirabayashi was also used: recovery rate (%) = (postoperative JOA score - preoperative JOA score)/(17 - preoperative JOA score)×100 [13]. Standard anterior–posterior, lateral and flexion-extension X-rays of cervical spine were obtained at every follow-up visit. MRI of cervical spine is also requested in the latest follow-up in this study, but some patients refused the procedure because they did not feel any discomfort or progression associated with their residual symptoms. The alignment of the affected segment (sagittal segmental alignment, SSA) was defined as the angle between the superior endplate and the inferior endplate of the fused segment on lateral radiographs, while the sagittal alignment of the whole cervical spine (SACS) was defined as the angle formed by the lines parallel to the posterior border of C2 and C7, respectively [14]. All values were repeated three times and the results were averaged, and the value was considered positive in lordosis and negative in kyphosis (<0°). Fusion was defined as the presence of the following features: 1) absence of radiolucent lines/area across the fusion site or around any of the screw sites; 2) presence of bridging trabeculae across the fusion site; 3) absence of motion between the spinous processes on flexion–extension X-rays [15]. If the fusion was questionable, it was confirmed by sagittal reconstructive CT scan. Graft/cage related complications including subsidence and migration were also recorded. TMC subsidence was defined as any migration of the TMC into the adjacent vertebral bodies more than 3 mm, and migration was defined as graft/cage beyond the leading edge of the upper and lower vertebral connection more than 3 mm on lateral radiographs [12]. Moreover, plate and screw related complications were also recorded.

Various factors that may affect the clinical outcome such as gender, age, preoperative duration of symptoms, duration of follow-up, preoperative JOA score, high-intensity signal on T2-weighted MRI, smoking status, combined cervical OPLL, number of segments involved, type of graft, intraoperative morphologic findings, loss of lordosis and graft/cage subsidence were all examined. Patients more than 60 years old were considered elderly; herniated soft discs were considered as intraoperative “soft" findings; posterior osteophyte, OPLL, calcified disc were considered as “hard"; duration of symptoms more than 12 months was categorized as long term; SACS ≤0° was considered as a loss of lordosis.

### Statistical Methods

The paired *t* test was used to detect changes in JOA score, SSA and SACS before surgery and at the last follow-up. The Mann-Whitney U test for independent samples was used to compare symptom resolution with continuous variables such as age, duration of symptoms before surgery, duration of follow-up and preoperative JOA score. The Chi-square test was used to compare symptom resolution with nominal variables, such as gender, smoking status, high-intensity signal on T2-weighted MRI, combined cervical OPLL, number of segments involved, type of graft, intraoperative morphologic findings, loss of lordosis and graft/cage subsidence. Furthermore, logistic regression analysis was conducted to determine the factors best correlating to symptom resolution. Analysis was performed using SPSS for Windows, Version 16.0 (SPSS, Chicago, IL), and a *P* value of less than 0.05 was considered significant.

## Results

### Patient Characteristics

A total of 93 males and 52 females completed the follow-up, with a mean age at operation of 51.0 years (range, 32–66 years). The mean follow-up was 102.1 months (range, 60–186 months). The average duration of symptoms before surgery was 28.4 months (range 0.25–180 months). High-intensity signal on T2-weighted MRI was found in 18 (12.4%) patients, and cervical OPLL was found in 22 (15.2%) patients. A smoking habit was found in 46 patients (31.7%) before surgery. (Table 1) The average preoperative JOA score was 10.7±1.8, while 20.7% of these patients had JOA score≤9. Cervical corpectomy was performed at 1 level in 133 patients (91.7%) and 2 levels in 12 patients (8.3%). Iliac-crest bone autograft was used for vertebral reconstruction in 28 patients (19.3%) and TMC filled with local vertebral bone autograft was used in 117 patients (80.7%). The intraoperative morphologic findings of spinal cord compression were “soft" in 105 patients (72.4%), “hard" in 15 patients (10.3%), and both “soft" and “hard" in 25 patients (17.2%). (Table 1)

**Table 1:** Summary of preoperative features and intraoperative findings.

	1-level corpectomy	2-level corpectomy
No. of patients	133	12
Male (%)	84 (63.2)	9 (75.0)
Age (years)	51.9±7.2	54.5±8.0
Symptom duration (months)	32.4±39.4	28.1±37.6
Follow-up (months)	102.4±26.5	99.1±19.8
High-intensity signal in spinal cord (%)	17 (12.8)	1 (8.3)
Smoking status	41 (30.8)	5 (41.7)
Combined with OPLL	20 (15.0)	2 (16.7)
Type of graft		
autograft	28 (21.1)	0 (0.0)
CTMC	105 (78.9)	12 (100.0)
Intraoperative morphologic findings		
Soft	96 (72.2)	9 (75)
Hard	14 (10.5)	1 (8.3)
Both	23 (17.3)	2 (16.7)

No., number; OPLL, ossified posterior longitudinal ligament; TMC, titanium mesh cage.

### Radiographic outcomes

**Figure 1:**
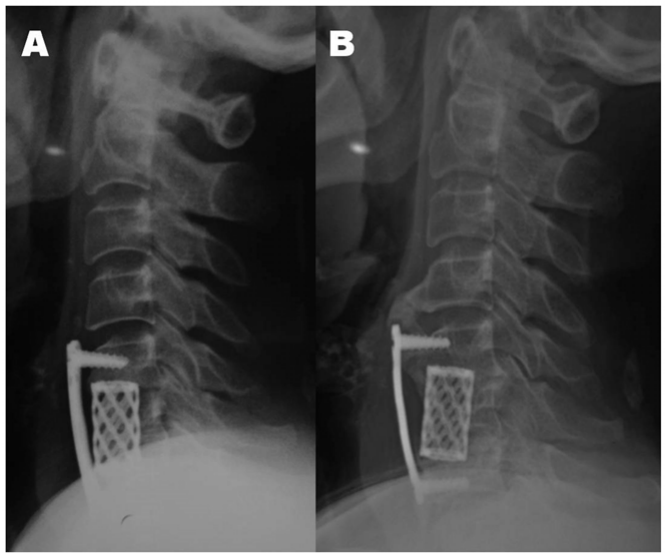
A 52-year-old female who underwent 1-level corpectomy and TMC fusion. **2A.** Postoperative lateral X-ray on the second day after operation showed detectable gap between anterior plate and upper vertebral body. **2B.** Postoperative lateral X-ray at the 5-year follow-up showed noticeable screw loosening at the upper vertebral body and spontaneous fusion of heterotopic ossification at the adjacent level.

The average SACS of these patients significantly increased in terms of cervical lordosis, which elevated from 15.9°±8.4° preoperatively to 22.5°±7.7° at the last follow-up (*P* = 0.000). Patients underwent 2-level ACCF procedure had more lordotic SACS than those underwent 1-level procedure postoperatively (*P* = 0.009). (Table 2) Loss of lordosis (SACS ≤0°) postoperatively was found in 7 patients (4.8%), and no patients were found with kyphosis (SACS<0°). The average SSA was 2.09°±3.5° preoperatively and 11.1°±4.6° postoperatively, which represented an average 9.1° lordotic increase (P = 0.000). (Table 3) TMC subsidence was identified in 13 patients (11.1%, 13/117). There was no difference between patients underwent 1-level and 2-level procedure (P = 0.463). Spinal fusion was noted in 96.2% of patients underwent 1-level procedure and 100% of those underwent 2-level procedure, thus the overall fusion rate was 96.6%. No significant difference in the fusion rate was observed between patients using iliac-crest bone autograft (96.4%) and TMC (96.6%) (*P* = 1.000). Graft/cage migration and screw loosening was found in 2 and 1 patient that underwent 1-level procedure, respectively. The patient with screw loosening had no further intervention because of the absence of symptoms and the spontaneous fusion of heterotopic ossification at the adjacent level. (Figure 1) No other plate and screw related complications were detected.

**Table 2:** Summary of pre- and postoperative SACS(°) in relation to the levels involved.

No. of levels	Preop	Last postop	Mean increase in SACS
1-level	15.4±8.6	22.0±7.8	6.6±2.6
C4	15.2±5.6	22.4±4.2	7.2±2.5
C5	15.6±9.2	22.2±8.4	6.6±2.7
C6	16.2±7.0	22.0±7.2	5.7±1.4
C7	−3.0±4.2	10.5±7.8	13.5±3.5
2-level	20.7±3.6	27.8±4.0	6.9±5.0
C4–5	19.6±4.1	26.4±2.9	6.6±4.9
C5–6	22.4±2.4	29.8±4.9	7.4±5.7

No., number; SACS, sagittal alignment of the whole cervical spine; Preop, preoperative; Postop, postoperative.

**Table 3:** Summary of pre- and postoperative SSA(°) in relation to the levels involved.

No. of levels	Preop	Last postop	Mean increase in SSA
1-level	1.6±3.0	10.7±4.3	9.1±2.6
C4	0.8±2.0	8.7±4.0	7.9±2.2
C5	2.3±3.2	11.5±4.3	9.3±2.6
C6	0.5±2.1	9.5±4.1	9.0±2.8
C7	−1.5±0.7	8.0±1.4	9.5±2.1
2-level	7.2±5.1	16.7±3.2	9.4±3.4
C4–5	4.3±4.5	15.9±3.8	11.6±2.5
C5–6	11.2±2.4	17.8±1.9	6.4±1.8

No., number; SSA, sagittal segmental alignment; Preop, preoperative; Postop, postoperative.

### Clinical outcomes and the Prognostic Factors

The overall mean JOA score was 10.7±1.8 preoperatively and 14.5±2.1 postoperatively, representing a mean 3.8±1.3 increase (*P* = 0.000). No improvement was observed in 5 patients (3.4%), and decline in JOA score during the follow-up period was noted in 33 patients (22.8%). The recovery rate was 62.5% in all patients, with 62.4% and 63.6% in patients underwent 1-level and 2-level procedure respectively. (Table 4) Recovery rate ≤50% (fair group) was observed in 31 patients and >50% (good group) in 114 patients. Univariate analysis revealed that there was no difference in gender, age, duration of follow-up, intramedullary signal change, smoking status, combined cervical OPLL, number of segments involved, type of graft and intraoperative morphologic findings between the two groups. However, patients with fair recovery rate had significantly longer duration of symptoms (*P* = 0.001) and lower preoperative JOA score (*P* = 0.000). Further logistic regression analysis showed that preoperative duration of symptoms >12 months (OR: 2.667, *P* = 0.05), high-intensity signal in spinal cord (OR: 3.581, *P* = 0.031) and preoperative JOA score ≤9 (OR: 4.836, *P* = 0.003) were important predictors of fair recovery rate.

**Table 4:** Summary of pre- and postoperative JOA score in relation to the levels involved.

No. of levels	No. of patients	Mean follow-up (months)	Preop JOA score	Last postop JOA score	Mean increase in JOA score
1-level	133	102.4±26.5 (60–186)	10.7±1.8	14.5±2.2	3.8±1.3
C4	14	114.3±32.4 (72–186)	10.7±1.7	13.9±2.3	3.1±1.5
C5	85	101.3±26.0 (60–178)	10.6±1.9	14.4±2.4	3.7±1.3
C6	32	99.5±24.6 (60–153)	10.8±1.7	14.8±1.6	4.0±1.2
C7	2	112.5±36.1 (87–138)	10.0±1.4	14.5±0.7	4.5±0.7
2-level	12	99.1±19.8 (60–130)	10.8±2.1	14.8±1.4	3.9±1.1
C4–5	7	95.3±21.1 (60–126)	10.3±2.1	14.4±1.5	4.1±1.1
C5–6	5	104.4±18.7 (86–130)	11.6±2.1	15.2±1.1	3.6±1.1

No., number; JOA, the Japanese Orthopedic Association; Preop, preoperative; Postop, postoperative.

### Adjacent segment disease and Repeated Surgery

**Figure 2:**
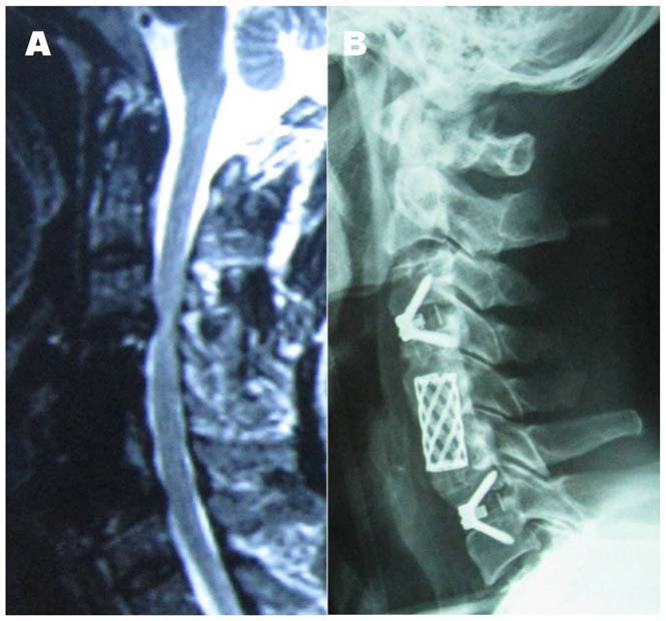
A 58-year-old male who received a reoperation for symptomatic adjacent segment disease. **1A.** Postoperative MRI at the 6-year follow-up showed degeneration at upper and lower discs adjacent to the fused segments. **1B.** Postoperative lateral X-ray on the second day after the second operation which performed by using Zero-P System (Synthes).

Symptomatic adjacent segment disease confirmed by clinical and MRI examination was found in 16 patients (11.0%), and all of these ASD patients had undergone 1-level ACCF. The main problem was chronic pain and/or numbness in upper extremity in 10 patients, unsteady gait and/or lower extremity weakness in five patients, and muscle atrophy in one patient. Repeated surgery because of ASD was performed in 7 (4.8%) cases, and the repeated surgery was conducted only when clear signs and symptoms of radiculopathy and/or myelopathy were confirmed. (Figure 2)

## Discussion

The strengths of this study are that the clinical and radiographic data with a minimum 5-year follow-up provides insights into the long term outcome of ACCF in treating patients with CSM, and nearly all known factors were included in the analysis for identifying the determinants of long term clinical outcome. In addition, the examinations during the follow-up were performed by investigators who had no therapeutic relationship to the individual patient. The present study revealed that ACCF is effective in treating patients with CSM, the mean JOA score at the last follow-up increased significantly when compared with the preoperative value. Long term radiographic follow-up showed fusion occurred in 96.6% of the patients. The results of ACCF in restoration of cervical segmental lordosis, TMC subsidence, graft/cage migration and screw loosening were also satisfactory. Patients with preoperative duration of symptoms >12 months, high-intensity signal in spinal cord and preoperative JOA score ≤9 may be at higher risk of fair recovery rate.

The natural history of CSM is mixed and unpredictable. Patients with CSM may present as a gradual decline in neurological function or in a progressive setting with a long period of quiescence [3]. Surgical intervention has been the mainstay for progressive CSM because conservative treatment had not been proved to be effective in altering the natural course of this disease. The principle goal of surgical intervention is to achieve adequate expansion of the canal diameter, thus providing the cord with adequate space to avoid static or dynamic compression. ACCF is an established treatment for cervical degenerative diseases, which offers several advantages, including direct decompression of neural structures, immediate stabilization of operated segments, solid fusion and maintenance or restoration of normal cervical alignment [6], [16].

According to the fusion criteria described above, an overall fusion rate of 96.6% was noted in the current study, which was similar with the previous reports [17], [18]. Non-fusion was observed in 5 patients, 3 of them had a smoking history, and none of them was symptomatic enough to require a revision surgery. Although it had been reported the fusion rate would decrease with the extension of the fused levels, non-fusion was not found in the patients underwent 2-level corpectomy which is mainly due to a small sample of the 2-level procedure population. A significant lordotic increase was observed in both SSA and SACS postoperatively compared with the preoperative data. Loss of lordosis of SSA and SACS was found in 5 and 7 patients respectively, and results showed a significant relationship between them (*P* = 0.008). It is reasonable that patients underwent 2-level procedure had more lordotic SACS than those underwent 1-level procedure because of the fixation of the longer pre-curved plate. Andaluz et al reported a 30.8% incidence of chronic neck pain during long term follow-up and 75% of which developped in patients with postoperative kyphosis. A significant relationship between postoperative C2–7 regional kyphosis and chronic neck pain was also found [18]. However, the incidence of chronic neck pain was 15.9% and there was no SACS kyphosis in the current study. As reported, the anterior plating system of less than 3 levels is helpful not only in maintaining appropriate cervical alignment but also in protecting the graft from dislodgement to work partially for fusions [19]. Migration was found only in two patients (1.4%) in this series, while the migration rate was reported as 6.4% in patients underwent ACCF without plate fixation [20].

As reported, deterioration could occur in any postoperative period. Decline in JOA score during the follow-up period in our study was noted in 22.8% of these patients, and most of which had slight declines without severe clinical symptoms. The mean RR in the current study was 62.5% after a minimum 5-year follow-up, and a good result (RR>50%) was observed in 78.6% of these patients, which is consistent with previous reports [10], [21]. Ikenaga et al assessed the long term results over 10 years of ACCF for Multilevel CSM by evaluating 31 patients, results showed an RR of 60.7% at 3 months after surgery and 56.4% at the last follow-up [10]. Xu et al evaluated 107 patients who underwent anterior surgery for CSM with more than 10 years of follow-up, and found the mean RR of the patients was 56.8%, and 70.1% had a good result with a RR>50% [21]. As noted by most investigators, there are multiple factors that play roles in the improvement of surgical treatment of CSM. Whether age is a negative prognostic factor for clinical outcome of anterior surgical treatment is still under debate. Matsuda et al studied 17 CSM patients older than 70 years and reported a lower peak recovery rate of 48.4% compared with 24 control patients younger than 65 years whose recovery rate was 69.4% [22]. Naderi et al had also reported that patients younger than 60 years had a better improvement after surgery than older patients [23]. However, the final JOA score was found to have no relation with age in Ikenaga's study [10]. Similarly, no difference in age was found between patients with fair and good results in our study. Intramedullary signal intensity change on MRI was also reported as an important factor that affects the outcome. Mehalic et al reported that patients who improved better clinically had less increased signal intensity on T2-weighted MRI in the cervical cord postoperatively, whereas patients who did not improve or worsened after surgery had the same or greater increased signal intensity [24]. In addition, Suri et al found that the presence of decreased signal intensity on T1-weighted MRI in addition to increased signal intensity on T2-weighted MRI of the spinal cord is predictive of a worse postoperative outcome than T2 changes alone [25]. It is also important to note that patients with increased signal intensity on T2-weighted MRI were older, had a longer duration of disease, which may explain why patients with increased signal intensity on T2-weighted MRI are prone to have worse postoperative functional status [26]. There have been several studies that provided multivariate analysis on the successful surgery treated patients, and the conclusions are similar. Patients may be successfully treated surgically if they have lower preoperative JOA scores, a canal expansion greater than 40% postoperatively, and a younger age [27], [28].

It is reported that cervical fusion increases the risk of subsequent symptomatic adjacent segment disease, which is estimated to affect 2.9% of patients per year [29]. However, whether adjacent level degeneration is the result of the fusion, or simply the progression of the natural deterioration of the motion segments, is still a matter of controversy. Walraevens et al assessed the long term results (4–8 year) of disk replacement surgery with the Bryan Cervical Disc in 87 patients, and 4 patients (4.6%) of them underwent repeated surgery because of ASD, which is similar with our results [30]. Thus, ASD is considered to occur more frequently in patients with preoperative evident adjacent disc degeneration than those with preoperatively intact discs. Moreover, even in patients with evident ASD, clinical symptoms remain scant and most of them can be resolved conservatively, which is also in accordance with previous findings [31].

The main limitation of this study is that this is a retrospective review with a high rate of loss to follow-up because of alterations in patient contact details such as address or phone number during the past 5–15 years. Secondly, there was no mandatory requirement for return visit during the 2–4 years follow-up, thus the consecutive data could not be obtained. Furthermore, MRI was performed in 47% of these patients at the last follow-up mainly due to the high cost; and some patients refused to do this because of the absence in discomfort or the slow progression of residual symptoms. We hope that future studies will achieve a higher follow-up rate and a series of consecutive data in long term follow-up research. Factors that affect the long term clinical outcome should be further evaluated in controlled clinical trial, such as high-intensity signal in spinal cord and long preoperative duration of symptoms. In addition, comparisons between ACCF and other techniques (such as ACCF vs ACDF, and ACCF vs laminoplasty) are also needed in the assessment of long term results of clinical outcome, fusion rate, cervical sagittal alignment, and adjacent segment disease.

In conclusion, after a minimum 5-year follow-up after ACCF with anterior plate fixation, clinical outcomes were satisfactory in terms of JOA score and the recovery rate. “Good" clinical outcome was achieved in 78.6% of these patients, and logistic regression analysis showed that preoperative duration of symptoms, intramedullary signal intensity change on T2-weighted MRI and preoperative JOA score were significantly related to the recovery rate. The correction of SSA and SACS were comparable with other published studies. Within our results, repeated surgery for ASD was necessary only in 4.8% of these patients. However, extra efforts should be made to improve the follow-up rate and a regular and consecutive follow-up is thus ideal.
